# Pervasive influence of idiosyncratic associative biases during facial emotion recognition

**DOI:** 10.1038/s41598-018-27102-z

**Published:** 2018-06-11

**Authors:** Marwa El Zein, Valentin Wyart, Julie Grèzes

**Affiliations:** 1 Laboratoire de Neurosciences Cognitives (Inserm unit 960), Département d’Etudes Cognitives, Ecole Normale Supérieure, PSL Research University, 75005 Paris, France; 2 Institute of Cognitive Neuroscience, University College London (UCL), WC1N 3AR, London, United Kingdom

## Abstract

Facial morphology has been shown to influence perceptual judgments of emotion in a way that is shared across human observers. Here we demonstrate that these shared associations between facial morphology and emotion coexist with strong variations unique to each human observer. Interestingly, a large part of these idiosyncratic associations does not vary on short time scales, emerging from stable inter-individual differences in the way facial morphological features influence emotion recognition. Computational modelling of decision-making and neural recordings of electrical brain activity revealed that both shared and idiosyncratic face-emotion associations operate through a common biasing mechanism rather than an increased sensitivity to face-associated emotions. Together, these findings emphasize the underestimated influence of idiosyncrasies on core social judgments and identify their neuro-computational signatures.

## Introduction

Our social life is punctuated by countless judgments about others, which determine first and foremost whether or not to engage in interactions with them. Does this person look angry? Can he or she be trusted? These different kinds of social judgments—from the recognition of a fleeting emotion, to the inference of stable personality traits—are influenced by facial cues displayed in others.

Indeed, past research has shown that facial morphological features reliably influence judgments of personality traits^1^, for example, ‘babyfaces’ are judged as weak and submissive, whereas masculine faces are perceived as dominant and aggressive. Such associations are *shared*—they are consistent across different individuals—and predict significant social outcomes such as political success^2^, even when they are inaccurate^1,3^. By contrast, some associations are *idiosyncratic*: they differ substantially across observers whilst remaining consistent for each observer. The degree of consensus across individuals may vary considerably when judging faces on a variety of personality traits including attractiveness^4–6^, intelligence, creativity and competence^6^.

Shared associations are not limited to facial morphology and personality, but extend also to emotion: certain morphological features appear to express emotions when in fact they are expressionless^7,8^ and may bias the perception of brief displays of emotion^9^—masculine faces are perceived as being angrier than babyfaces expressing the same emotion^1^. Yet the extent to which emotion recognition is influenced by *idiosyncratic* associations remains unknown. In the current study, we aim first to investigate the specific contribution of such idiosyncrasies to emotion recognition; second, to evaluate the time scale under which these idiosyncrasies emerge; and third, to characterise their computational and neural mechanisms.

To address these questions, we asked participants to categorise ambiguous facial expressions of emotion as anger or fear. To measure shared and idiosyncratic associations between facial morphology and emotion (aim 1), we added a post-test at the end of the main emotion categorisation task. Each participant rated each face seen during the main task, shown with a neutral expression, according to how frequently he/she thought it expressed anger or fear during the main task. This post-test was used to dissociate idiosyncratic associations from shared associations.

To determine the time scale under which idiosyncrasies might emerge (aim 2), we induced arbitrary and incidental pairings between particular faces and emotions: each face was systematically presented expressing one specific emotion at the beginning of each experimental block (half anger, half fear). This manipulation aimed at testing whether idiosyncrasies vary on a short time scale–in which case they should match the *induced* arbitrary face-emotion associations, or reflect stable inter-individual differences in face-emotion associations – in which case they should be independent of them.

Finally, to identify neural and computational mechanisms underlying face-emotion associations (aim 3), we analyzed human behavior and electrical brain activity using electroencephalography (EEG) during emotion categorisation in a second group of subjects using the same facial stimuli. Using a canonical decision-theoretic framework, we tested whether facial morphology triggers either a shift in decision criterion toward the emotion associated with the face, or an increased sensitivity to the facial cues characteristic of the emotion associated with the face.

## Results

### Dissociation of induced, shared and idiosyncratic face-emotion associations

**Figure 1:**
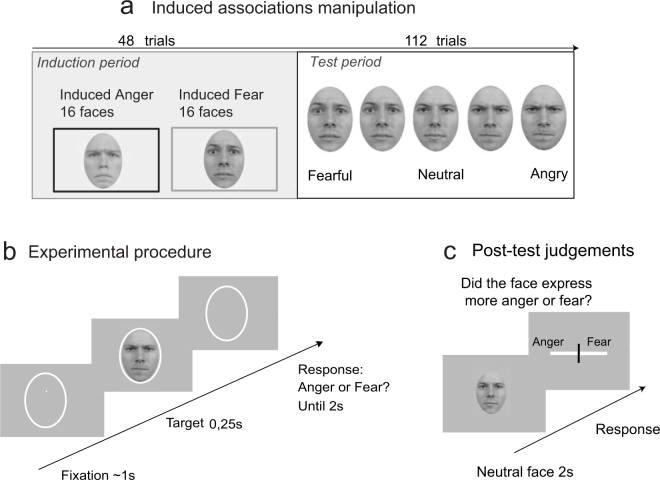
Stimuli and experimental procedure. **(a)** Left panel, the first 48 trials of each block consisted of faces expressing either anger or fear at the highest level of emotion, to manipulate expressions of individual faces. Right panel, an equal number of morphed anger and fear expressions were presented in the other 112 trials of the block, examples are shown for one face: morphs from neutral to fearful/angry expressions providing evidence for one or the other emotion. **(b)** Following fixation, a facial expression is presented for 250 ms, after which the participant indicates whether the face expressed anger or fear within 2 seconds. No feedback is provided after response. **(c)** At the end of the experiment, participants performed a post-test in which they rated on a scale whether they think each neutral face (that appeared for 2 sec) expressed more anger or fear during the experiment. The faces shown in the figure are taken from the Radboud Faces database^32^.

In the main emotion categorization task, participants categorized 32 different faces as expressing anger or fear. Emotion strength was manipulated by presenting ‘morphed’ facial expressions ranging from neutral to intense anger or fear. To induce arbitrary face-emotion associations, each experimental block started with a short ‘induction’ period. During this period, we randomly paired, differently for each participant, half of the 32 faces with anger only (16 randomly selected faces) and the other half with fear only (the remaining 16 faces). Both emotional expressions were presented with the highest level of emotion (Fig. 1a). Faces arbitrarily associated with anger are labeled ‘Induced anger’, and those arbitrarily associated with fear are labeled ‘Induced fear’. This short induction period was directly followed by a ‘test’ period during which all 32 faces expressed an equal number of angry and fearful expressions at variable emotion strength. In both the ‘induction’ and ‘test’ periods, participants had to categorize the facial expression that appeared on the screen for 250 milliseconds (ms) as expressing anger or fear (Fig. 1b), with no explicit warning about the transition between the ‘induction’ and ‘test’ periods.

After the main task, participants performed a ‘post-test’ rating task, during which the same 32 faces were presented, but this time with a neutral expression. Participants were requested to rate each face in terms of how frequently they think this specific face expressed anger or fear during the main task (Fig. 1c). Post-test ratings were used to measure and dissociate induced, shared and idiosyncratic face-emotion associations. We first observed that induced associations were not reflected in the post-test. Indeed, neither the difference between ‘induced anger’ and ‘induced fear’ mean ratings (T_30_ = 0.56, p = 0.57, paired t-test -Bayes Factor = 0.29), nor the area under the receiver operating curve (ROC) were significant (0.504 ± 0.003, t-test against chance level, T_30_ < 0.3, p > 0.8). These results suggest that participants were unable to identify induced arbitrary face-emotion associations during the post-test.

Next, we used the post-test ratings to define orthogonal shared and idiosyncratic face-emotion associations using a ‘leave-one-out’ procedure (see Methods for details). On one hand, we defined ‘shared anger’ and ‘shared fear’ as those faces appearing respectively more angry or fearful to the entire group of participants (mean rating provided for each face by all participants). On the other hand, ‘idiosyncratic anger’ and ‘idiosyncratic fear’ corresponded to faces appearing respectively more angry or more fearful to a particular participant than to other participants (i.e., difference between an individual participant’s rating and the mean rating provided for the same face by the group of participants).

We then assessed the relationship between induced face-emotion associations and these shared and idiosyncratic associations. Induced associations did not correlate with shared associations (*T*_1,30_ = 0.32, p = 0.74, t-test against zero), nor idiosyncratic associations (*T*_1,30_ < 0.01, p = 1, t-test against zero). This indicates that the three types of face-emotion associations (induced, shared and idiosyncratic) are independent. Importantly, this also shows that idiosyncratic associations estimated in the post-test cannot be explained only by short-term experience (i.e. induced associations).

### Quantifying emotion recognition biases triggered by shared, idiosyncratic and induced face-emotion associations

**Figure 2:**
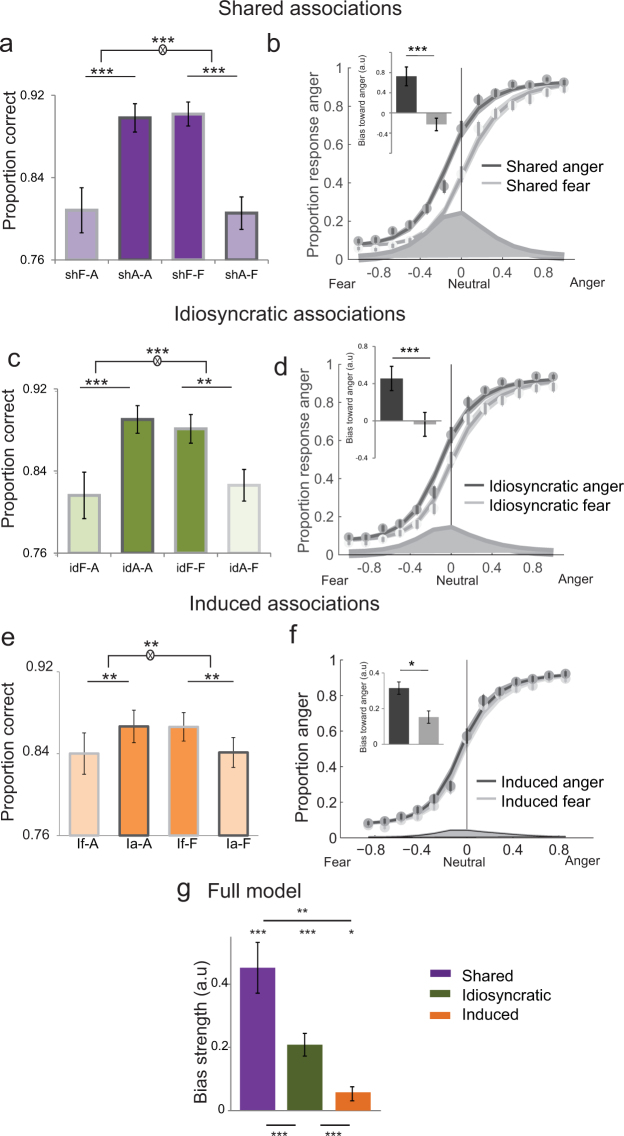
Influence of shared, idiosyncratic and induced associations on emotion recognition. **(a)** Proportion of correct responses as a function of shared associations (from left to right) Shared Fear-Anger (meaning Shared Fear faces expressing Anger), Shared Anger-Anger, Shared Fear-Fear and Shared Anger-Fear. Congruent combinations between facial traits and emotions are highlighted in dark. **(b)** Psychometric function representing the proportion of ‘anger’ responses as a function of the evidence for anger (proportion morph, 0 for neutral, negative towards fear, and positive towards anger) for Shared anger faces (dark grey) and Shared fear faces (light grey). Dots and attached error bars indicate the human data (mean ± s.e.m.). Lines and shaded error bars indicate the predictions of the best-fitting model. **(c)** Proportion of correct responses as a function of idiosyncratic associations (from left to right) Idiosyncratic Fear-Anger, Idiosyncratic Anger-Anger, Idiosyncratic Fear-Fear and Idiosyncratic Anger-Fear. **(d)** Same as (b) for Idiosyncratic anger faces and Idiosyncratic fear faces. **(e)** Proportion of correct responses as a function of induced associations (from left to right) Induced fear-Anger, Induced anger-Anger, Induced fear-Fear and Induced anger-Fear. **(f)** Same as (b) for faces belonging to Induced anger and those belonging to Induced fear. **(g)** Estimated decision bias strength toward congruent face-emotion associations for the model integrating all three sources of social biases: shared (violet), idiosyncratic (green) and induced (orange) associations. ^*^p < 0.05, ^**^p < 0.01, ^***^p < 0.001.

We looked at the effect of these three types of face-emotion associations on emotion recognition during the main task. First, shared associations interacted with the displayed emotion on the accuracy of emotion recognition (*F*_1,30_ = 207.3, *p < *0.001, ANOVA), with ‘shared anger’ faces better recognised as angry as compared to ‘shared fear’ faces and ‘shared fear’ faces better recognised as fearful than ‘shared’ anger faces (Fig. 2a).

To characterise how shared associations impact emotion recognition, we compared different decision-making models (inspired by signal detection theory) that instantiate the potential mechanisms by which these associations influence emotion recognition. Shared associations can either trigger a *bias* toward anger or fear, or alternatively modulate the perceptual *sensitivity* to these emotions. While the two accounts predict similar effects on recognition accuracy (i.e. increased performance for anger in ‘shared anger’ faces and fear in ‘shared fear’ faces), a bias effect (shift toward anger responses for ‘shared anger’ faces) would be maximal for neutral expressions, whereas a sensitivity effect (increased for anger-signifying cues in a ‘shared anger’ faces) would be maximal for intermediate levels of emotion. We compared a model where shared associations bias the responses toward ‘congruent’ conditions (anger in ‘shared anger’ faces, and fear in ‘shared fear’ faces) (model 1), to a model where there is an increase in sensitivity for these congruent conditions (model 2). Bayesian model selection combined with a cross-validation procedure to estimate the log-evidence of the different models showed that the effect was better explained by a change in the decision bias (model 1) toward congruent combinations (‘shared anger’/anger, ‘shared fear’/fear), when compared with an increased sensitivity to these combinations (fixed-effects Bayes factor ≈ 10^52.7^, random-effects *p*_exc_ = 0.99) (Fig. 2b).

Second, we looked at the influence of idiosyncratic associations that reflect each participant’s unique associations between certain faces and emotions, independently from shared associations (using the same approach as above and described in Methods). Idiosyncratic associations also interacted with the displayed emotion on the accuracy of emotion recognition (*F*_1,30_ = 41.5, *p* < 0.001, ANOVA) during the task: faces belonging to ‘idiosyncratic anger’ were better recognised as angry when compared to faces belonging to ‘idiosyncratic fear’, and faces belonging to ‘idiosyncratic fear’ were better recognised as fearful when compared to faces belonging to ‘idiosyncratic anger’ (Fig. 2c). The effect was mediated by a change in decision bias toward congruent face-emotion associations, rather than sensitivity (fixed-effects Bayes factor ≈ 10^9.0^, random-effects *p*_exc_ = 0.96) (Fig. 2d).

Third, we assessed the effect of induced associations on emotion recognition. Induced associations interacted with the displayed emotion on the accuracy of emotion recognition (*F*_1,30_ = 7.71, *p* = 0.009, ANOVA). Faces belonging to ‘induced anger’ and ‘induced fear’ (shown as angry or fearful in the induction phase) were better recognised as angry and fearful, respectively, as compared to those belonging to ‘induced fear’ and ‘induced anger’ (Fig. 2e). Once more, model selection indicated that the effect was mediated by a change in decision bias toward congruent face-emotion associations, i.e., induced anger showing an angry expression and induced fear showing a fearful expression (fixed-effects Bayes factor ≈ 10^5.3^, random-effects *p*_exc_ = 0.87) (Fig. 2f).

### Persistence of idiosyncratic face-emotion associations

To assess the specific contribution of idiosyncratic associations, irrespective of both shared and arbitrarily induced associations, we accounted for all the observed biases simultaneously and quantified their respective contributions in one single behavioral model. All three sources of biases additively influenced participants’ behavior during emotion recognition: the bias that reflected participants’ idiosyncratic associations (T_30_ = 5.80, p < 0.001, t-test against zero), the bias related to shared associations (T_30_ = 5.63, p < 0.001, t-test against zero), and the bias related to induced associations (T_30_ = 2.58, p = 0.01, t-test against zero) (Fig. 2g – also see Supplementary Figure 1 showing individual variability for each of the biases). Idiosyncratic and shared biases were both much stronger than the induced bias (idiosyncratic vs. induced: T_30_ = 3.34, p = 0.002, paired t-test; shared vs. induced: T_30_ = 4.57, p < 0.001, shared vs. idiosyncratic: T_30_ = 3.65, p < 0.001, paired t-test). Moreover, the full model better fitted the data as compared to models not accounting for idiosyncratic biases (pexc > 0.94, Bayes factor > 10^23.6^). Finally, the full model—fitted separately in the first and second part of the experiment—showed that none of the three types of biases triggered by idiosyncratic, shared and induced associations varied in time (all T < 1.42, all p > 0.16, paired t-test). On one hand, this result shows that adaptation effects cannot account for the weakness of induced associations: indeed, their influence on emotion recognition did not increase nor decrease throughout the experiment. On the other hand, and more importantly, this result shows that idiosyncratic biases did not emerge gradually throughout the experiment: they rather seem to represent stable biases, unique to each individual. Together, these results show that biases in emotion recognition shared across human observers coexist with large and stable idiosyncrasies that cannot be explained by short-term experience with particular faces (i.e., our induction manipulation).

### Neural signature of shared associations in emotion recognition

In a previous independent electroencephalography (EEG) experiment, 24 healthy individuals performed exactly the same experimental task—recognition of morphed expressions of anger or fear (Fig. 1b)—without the induced face-emotion associations blocks or the post-test ratings on neutral faces^10^. To assess whether shared associations are represented in the brain along with emotion strength, and in the absence of a post-test rating task, we defined shared face-emotion associations directly from decisions in the emotion recognition task. For each face, ‘shared associations’ corresponded to the mean decision bias for that face across participants (see Methods).

**Figure 3:**
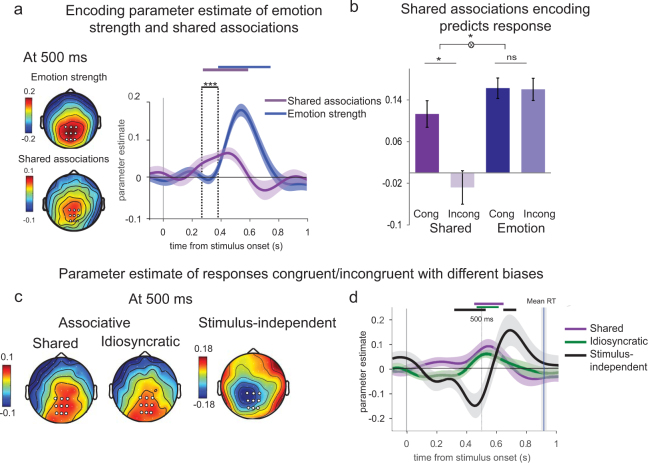
Neural signature of shared and idiosyncratic face-emotion associations. (**a)** Left panel: Scalp topography of encoding parameter estimate at 500 ms for emotion strength (top) and shared associations (bottom) expressed as mean parameter estimates in arbitrary units (a.u.). Right panel. Time course of shared associations (violet) and emotion strength (blue) encoding at parietal electrodes (indicated by white dots on the scalp topography). Shaded error bars indicate s.e.m. Thick violet and blue lines indicate significance against zero at a cluster-corrected p-value of 0.05. Encoding latency is significantly different between shared associations and emotion encoding. **(b)** Encoding of emotion strength and shared associations separately for conditions where participants responded congruently (cong) or incongruently (incong) with shared associations. **(c)** Scalp topographies at 500 ms after stimulus onset showing the contrast in arbitrary units (a.u) between trials where responses were congruent or incongruent with (from left to right) shared, idiosyncratic (stimulus-dependent/associative) and stimulus-independent biases. **(d)** Time course of the contrast between trials where responses were congruent or incongruent with associative shared (violet), idiosyncratic (green), and motor stimulus-independent (black) biases at parietal electrodes (indicated by white dots on the scalp topographies in (c)). The vertical black line shows mean reaction times across participants with shaded errors bar indicating s.e.m. ^*^p < 0.05, ^***^p < 0.001.

Instead of computing event-related averages, our EEG analyses consisted of a parametric regression-based approach where single-trial EEG signals were regressed against variables of interest (here the strength of the displayed emotion and shared associations) at each electrode and time point following the presentation of the face^10–12^. The resulting time course at each electrode represents the degree to which EEG activity ‘encodes’ (co-varies with) the emotion strength or shared associations. Emotion strength was encoded at 500 ms in centro-parietal EEG signals, known to encode the decision variable^11–13^ (T_23_ = 4.7, p < 0.001, t-test against 0). Shared face-emotion associations were also encoded at the same electrodes (Fig. 3a, left panel). Yet, besides being represented in decision signals, shared associations were processed about 100 ms before the emotion strength itself (Fig. 3a right panel) (shared face-emotion associations: 270 ms, emotion strength: 390 ms, T_23_ = 4.0, p < 0.001, see Methods for details). This earlier encoding is consistent with a decision bias in emotion recognition, thus corroborating the identified behavioral mechanisms underlying the influence of shared associations on emotion recognition.

Moreover, the encoding of shared associations was significant only in trials where subsequent responses were congruent with the shared emotion association for the presented face (T_23_ = 2.7, p = 0.01, paired t-test), thus showing it could predict upcoming responses (Fig. 3b). To verify whether this effect merely reflects trials where participants generally paid more attention to the face and thereby processed any facial information more accurately, we compared the encoding of emotion strength by splitting trial with responses congruent or incongruent with shared associations. We found no difference in the encoding of emotion strength when comparing these two types of trials (T_23_ = 0.09, p = 0.92, paired t-test). Furthermore, a significant interaction between the type of information encoded (emotion strength or shared associations) and the direction of the upcoming response (congruent or incongruent with shared associations) (F_1,23_ = 6.1, p = 0.02, ANOVA) (Fig. 3b) confirmed that the neural encoding of shared associations selectively influenced the direction of upcoming response.

### Common neural correlates of decision biases related to shared and idiosyncratic face-emotion associations

Results from our main behavioral experiment suggest that both shared and idiosyncratic face-emotion associations bias emotion recognition through a similar mechanism. These findings raised the question of whether the neural correlates for shared and idiosyncratic associations were also similar.

For the EEG experiment, we defined orthogonal shared and idiosyncratic face-emotion associations directly from decisions in the emotion recognition task. In other words, ‘shared anger’ and ‘shared fear’ faces represented faces that biased decisions respectively toward anger and fear for the group of participants. ‘Idiosyncratic anger’ and ‘idiosyncratic fear’ corresponded to faces that biased decisions toward anger or fear for a particular participant as compared to other participants (i.e., difference between the bias of a given participant for a face and the mean bias for the same face by all other participants) (see Methods).

We created two ‘shared’ and ‘idiosyncratic’ regressors (orthogonal by definition, given the construction of ‘shared’ and ‘idiosyncratic’ labels), which tagged single trials in terms of shared and idiosyncratic associations respectively, and the response provided by the participant – either congruent or incongruent with the association. We then assessed whether centro-parietal EEG signals at 500 ms differed between trials with association-congruent and -incongruent responses, for both shared and idiosyncratic associations. To do so, we performed a regression of EEG signals against these two regressors. We observed increased centro-parietal activity before the responses that were congruent with shared associations (T_23_ = 3.4, *p = *0.002, paired t-test at 500 ms) and idiosyncratic associations (T_23_ = 2.95, *p = *0.007, paired t-test at 500 ms) (Fig. 3c,d). This result indicates the additive influence of both types of associations on emotion recognition. The influence of shared and idiosyncratic face-emotion associations thus relies on similar neural correlates: an increased activity in decision signals preceding responses congruent with the observer’s face-emotion associations.

Interestingly, it is known that other types of decision biases, such as ‘response’ or ‘status quo’ biases, are usually associated with decreased decision signals preceding congruent responses^14,15^. In our emotion recognition task, the same analyses were performed on overall, face-independent decision biases - i.e. response biases toward responding ‘anger’ or ‘fear’. Decreased parietal activity at 500 ms was observed for responses congruent with the face-independent ‘response’ bias (*T*_23_ = 2.1, *p* = 0.04, paired t-test) (Fig. 3c,d). The topographies at 500 ms (Fig. 3c) and the time course (Fig. 3d) of the main effect of activity with/against different types of biases underline a common mechanism for shared and idiosyncratic face-emotion biases – i.e., stimulus-dependent associative biases, as opposed to stimulus-independent biases.

### Shared associations correspond to social attributes of dominance and trust

Particular facial traits such as masculine/feminine or baby-faced traits have been associated with higher order social attributes^1^. To assess whether shared associations between faces and emotional expressions of anger and fear are linked to particular social attributes, we conducted another rating task on a new independent sample of twenty healthy participants (10 females, mean age: 22.7 ± 0.6 years). Participants were requested to rate each of the faces used in the previous experiments, displayed with a neutral expression, in terms of dominance and trustworthiness traits. Faces were presented for 2 seconds, followed by the appearance on the screen of 2 continuous scales (going from “not at all” to “very much”) accompanied by the instructions: “How much is this face dominant/trustworthy?”. We took the mean ratings across participants for each face and correlated these ratings with shared associations obtained from the post-test in the main behavioral experiment.

First, dominance ratings negatively correlated with trustworthiness ratings (r = −0.49, p = 0.002, Pearson correlation). Second, the shared component of the post-test rating (mean rating across participants for each face) positively correlated with dominance (r = 0.55, p = 0.001, Pearson correlation) and negatively correlated with trustworthiness (r = −0.43, p = 0.01, Pearson correlation). Doing a similar correlation in the EEG experiment showed that shared associations (mean bias across participants for each face) positively correlated with dominance (r = 0.64, p < 0.001, Pearson correlation) and negatively correlated with trustworthiness (r = −0.43, p = 0.008, Pearson correlation). These highly significant correlations suggest that when faces are associated with anger and fear in a way that is shared across participants, these faces are also associated with dominance and trustworthiness. However, the precise direction of the effect (e.g. dominance associated with anger and trustworthiness associated with fear) is difficult to assess because the emotion recognition task consisted of a binary choice between anger and fear. We can only conclude that in such a context, the more dominant or untrustworthy a face is, the more it is recognised as angry, and the more trustworthy or non-dominant another face is, the more it is recognised as fearful.

## Discussion

Past research has shown that potentially unreliable^1,3^, yet pervasive, social judgments about others arise from facial morphology whose significance is similar across observers^1^. These shared associations have been demonstrated not only between specific facial morphology and social attributes (e.g. personality traits), but also between specific facial morphology and emotional expressions^9^. Here we show that humans also feature a large degree of *idiosyncrasy* in emotion judgement – that is, variation unique to each observer. A large part of this idiosyncratic component appears to be independent from short-term experience (in our study, arbitrarily associations induced at the beginning of each block). Using computational modeling based on canonical decision theory^16,17^, we identified a single mechanism responsible for both shared and idiosyncratic facial determinants of emotion recognition: a shift in decision criterion toward bias-congruent interpretations of facial cues. Finally, the influence of shared and idiosyncratic associations in emotion recognition was associated with a common signature in EEG activity – which differed from the signature of classical response biases in decision-making. Together, these findings highlight the underestimated influence of idiosyncratic associative biases on core emotional judgments.

By means of computational modeling of human decision-making, we characterised the specific mechanism by which facial morphology influences emotion recognition. Two main classes of mechanisms could explain the observed influence: a shift in decision criterion toward the emotion associated with identity-characteristic features – e.g., anger for a masculine face, or an increase in perceptual sensitivity to emotional cues associated with identity-characteristic features – e.g., anger-signifying cues such as lowered eye brows in the same masculine face. Here, by varying emotion strength parametrically, we could differentiate between these two mechanisms in choice behavior. We revealed that idiosyncratic and shared associations jointly influenced emotion recognition through a criterion shift – in contrast to other known contextual effects in emotion recognition such as gaze direction^10^. A criterion shift is generally thought to be less costly than an enhancement of perceptual sensitivity – which requires increasing the signal-to-noise ratio of neural processing. Face-emotion associations might rely on flexible shifts in decision criterion because such social judgments are often inaccurate^3^ and thus prone to many adjustments at various time scales – e.g., cultural (different cultures have different social codes) or contextual (different moods at work/home).

Our findings further support an automatic processing of facial cues constitutive of someone’s identity during emotion recognition. While traditional views emphasised an independent processing of emotion and facial identity^18,19^, there is now growing evidence in favor of a reciprocal interaction between emotion- and identity-characteristic facial attributes^20–22^ suggesting that both are processed automatically. The regression of emotion- and identity-characteristic features against electrical brain signals revealed that the encoding of identity-characteristic facial attributes in centro-parietal correlates of the decision variable^12,13^ emerged 100 ms earlier (270 ms) than the encoding of the emotion-characteristic features (390 ms). The timing of these effects is in line with previous reports of an early processing of facial features (starting at 170 ms) and a later processing (after 300 ms) of task-specific facial information^23,24^. The earlier encoding of facial morphological features in the decision process provides supplementary evidence in favor of a criterion shift in emotion recognition.

In addition, increased centro-parietal activity was observed prior to responses congruent with the face-emotion associations – whether shared (predictable across observers) or idiosyncratic (unique to each observer). The fact that shared and idiosyncratic biases rely on the same neural correlates supports the hypothesis of a single mechanism that might, speculatively, reinforce associative biases in social judgments irrespective of their fitness. The additional observation that the expression of both shared and idiosyncratic biases co-varies positively with the decision neural signal is also important. Indeed, this common directionality suggests that a stronger processing of identity-characteristic facial attributes increases the likelihood of the expression of corresponding bias in the subsequent response. This neural signature of ‘associative’ biases is opposite to those described for other decision biases – e.g., ‘response’ or ‘status quo’ biases^14,15^, which are independent of the stimulus. Their expression is typically associated with *decreased* decision signals preceding response. An important difference between associative and other kinds of biases is that only the first depends on the processing of stimulus attributes, and thus on the allocation of attentional resources to stimulus processing – here the identity-characteristic attributes of the presented face.

Shared associations were related to social attributes (dominance, trustworthiness). This finding supports previous results showing that social attributes such as trustworthiness influence emotion recognition^9^. Interestingly, dynamic facial expression and movements can camouflage facial morphology information when conversely judging trustworthiness, dominance and attractiveness^25^. The bi-directionality of interactions between emotional expressions (variable traits) and facial morphology (invariant, stable traits) suggests that the facial features at play in social attributions (such as attractiveness and trustworthiness) are partially overlapping with those used in emotion recognition.

In line with previous studies^4–6^, our findings draw attention to the existence and importance of idiosyncrasies in social judgments. Importantly, they extend previous observations of idiosyncrasy in judgments of personality traits (such as attractiveness^4–6^) to emotion recognition from faces. Such idiosyncrasies can stem from inter-individual difference in their associations between identity-characteristic facial features and emotions, their internal representations of emotional expressions^26^ (i.e. the model of an angry face for each observer) or their strategies for feature sampling in faces (i.e. one observer focuses on the mouth while another person focuses on the eyes to make emotion judgments). Although the present experiment cannot disentangle these potential sources of idiosyncrasy, it allowed us to evaluate the time scale under which they emerged. In agreement with inter-temporal idiosyncrasies, i.e. inconsistent social judgments for the same faces by the same observer^27–29^, our results show that short-term experience (induced associations) impacts emotion judgment from faces. Yet, a large degree of idiosyncrasy appears to reflect stable individual differences (independent from short-term induced associations and stable over the course of the experiment). These idiosyncrasies might reflect the unique life experience^30^ or environment^4^ of each observer, and could be maintained through generalization to other faces resembling that face^31^.

To conclude, our findings provide the mechanism by which face-emotion associations influence emotion recognition. They extend previous studies^4–6^ by revealing large idiosyncrasies during core emotion judgments – a factor which is only rarely taken into account when studying emotion recognition. Importantly, idiosyncrasies in social judgments have the advantage of avoiding strong and potentially inaccurate consensus during collective decisions, by adding variability in social judgments across observers. Future studies should directly address the impact of idiosyncrasies on collective decision-making and the emergence of consensus between individuals. Finally, the criterion shift by which face-emotion associations influence emotion recognition is known to be particularly flexible, and thus minimizes the biological cost associated with overcoming inaccurate social attributions on the basis of disconfirmatory evidence (e.g., social interactions) in a rapid and adaptive fashion.

## Methods

In this section, we describe the methods of our current experiment, as well as the methods from one of our previous electroencephalography experiments^10^ that allowed tackling the neural signature of the behavioral effects observed in the current experiment.

### Participants

#### Current Experiment

Thirty-one healthy participants (15 females; mean age: 22.3 ± 0.5 years) participated to the experiment.

#### Electroencephalography (EEG) experiment

Twenty-four healthy participants (12 females; mean age: 22.7 ± 0.7 years) participated to the experiment^10^.

All participants were right-handed, with normal (or corrected-to-normal) vision and no neurological or psychiatric history. All experiments were approved by the local ethics committee (Comite de Protection des Personnes, Ile-de-France VI, Inserm approval #C07–28, DGS approval #2007-0569, IDRCB approval #2007-A01125-48). Our studies were performed in accordance with the Declaration of Helsinki. All human participants provided informed written consent prior to the experiment according to institutional guidelines and regulations of the local research ethics committee.

### Stimuli

Stimuli consisted of faces adapted from the Radboud Faces Database^32^ that varied in emotion (neutral, angry or fearful expressions). The strength of emotional expressions varied with 7 levels of anger and 7 levels of fear equalized in perceived emotional strength and a neutral condition (see Fig. 1a for examples of stimuli). A complete description of the stimuli is provided in El Zein *et al*., 2015^10^. In the published EEG study, 36 faces (18 females) were used while in the current behavioral study, only 32 (16 females) out of these 36 faces were used only to reduce the length of the task that now included additional trials and a post-test that will be described in the experimental procedure below.

### Data availability

The datasets generated and analysed during the current study are available at this link: https://drive.google.com/drive/folders/1UjHf17poDLWoSMY0pXZ2bZv9Ai2mrzj1?usp=sharing.

## Experimental Design and Statistical Analysis

### Experimental design

#### Current Experiment

The task consisted of categorizing the faces as fearful or angry (Fig. 1b). Using the Psychophysics-3 Toolbox^33,34^, faces appeared for 250 ms on a black screen, after which participants had to give their response by pressing one of the two “ctrl” buttons localized on the keyboard with their right or left index (a maximum of 2 seconds to respond before the next trial). The faces shown at each trial covered the participant’s central vision and were displayed with a visual angle of 6.5 degrees. An Anger/Fear mapping was used (e.g Anger: Left hand, Fear: Right hand) kept constant for each subject, counterbalanced over all subjects.

To experimentally manipulate face-emotion associations, the 48 first trials of each block consisted of biased trials (‘induction period’): instead of each face expressing at an equal amount anger or fear, half of the faces expressed only anger (faces belonging to “induced anger”), and the other half expressed only fear (faces belonging to “induced fear”) (Fig. 1a). The assignment of faces to ‘Induced anger’ and ‘Induced fear’ varied from a participant to another, with the constraint that all faces were shown as expressing anger or fear across all participants. To maximise the chance of inducing the associations, only the highest level of emotion strength (7) was presented in these trials, and each stimulus was repeated 3 times. Importantly, the task during these blocks was unchanged, and participants had no explicit information about the content of the trials, they were only informed that the task might seem a little easier at the beginning of the blocs. Participants were indeed very accurate (95.5 ± 0.7%) during the ‘induction period’. Importantly, we chose to induce face-emotion pairings at the beginning of the blocks, and not during all the experiment, to avoid recency effects.

In the rest of the block ‘test period’ (112 trials), we presented levels 1 to 6 from the morph continuum of both emotions in an unbiased fashion (equal number of anger and fear for each face) as well as the neutral stimuli. The highest level of emotion, used in the ‘induction period’, was excluded from the ‘test period’. After each block, the percentage of correct responses was calculated from the ‘test period’ only and was shown to the participants to keep them motivated. The experiment was divided in 8 experimental blocks, 16 faces were manipulated in the first 4 blocs, and 16 different faces were manipulated in the 4 last blocks. All analyses were performed on only trials from the ‘test’ period. Note however that there were 2 outliers among the 32 faces with average classification errors of 27% error and 18% in the induction period (they were still recognized above chance). Removing them from the analysis did not change any of the reported results, neither qualitatively nor in terms of statistical significance.

At the end of the experiment, participants performed a post-test, during which they saw all the 32 faces encountered in the main experiment, but with a neutral expression. Each face appeared on the screen for 2 seconds and participants had to rate it on a scale (unlimited time) measuring the degree to which they thought that each face expressed anger or fear during the experiment (Fig. 1c). The position of anger and fear at the right and left of the scale was counterbalanced across participants.

#### EEG experiment

Faces appeared for 250 ms on a black screen, after which participants had to categorise the expressed emotion as anger or fear by pressing one of two buttons located on two external devices held in their right and left hands (Fig. 1b). An Anger/Fear mapping was used (e.g Anger: Left hand, Fear: Right hand) kept constant for each subject, counterbalanced over all subjects. Here, face-emotion associations were not manipulated; and no post-test was performed at the end of the experiment.

### Behavioral data analyses

#### Post-test analyses

To check whether the post-test reflected experimentally induced face-emotion associations, a two-tailed student t-test was conducted on the mean ratings for faces belonging to Induced anger and those belonging to Induced fear (considering the associations for each participant, as they differed across participants). The area under the receiver operating curve (AUC ROC) was also calculated to assess if the ratings were significantly different from chance or reflected the experimental manipulation.

Post-test ratings of neutral faces were employed to determine shared and idiosyncratic face-emotion associations. In practice, to label each face with respect to ‘shared’ and ‘idiosyncratic’ categories, we implemented a ‘leave-one-out’ procedure. First, we assessed whether the ratings provided by a participant for the different faces correlated positively with the mean ratings provided by all other participants for the same faces using the following linear regression:

1 $$rating({\rm{face}}=i,{\rm{subj}}=j)=\beta \,\times \,{{\rm{mean}}}_{{\rm{subj}}}[rating({\rm{face}}=i,{\rm{subj}}\ne j)]+\,\varepsilon $$

with ${\rm{face}}=i$ corresponding to face number i, ${\rm{subj}}=j$ to participant number j, ${{\rm{mean}}}_{{\rm{subj}}}[\,\,]$ signifying a mean across participants.

This analysis revealed a positive correlation coefficient $\beta$ for all participants (T_30_ = 9.18, p < 0.001, t-test against zero), indicating that ratings were consistent across participants. It remained significant when induced associations were modeled as an additional predictor of ratings (T_30_ = 9.31, p < 0.001, t-test against zero), suggesting that shared and induced associations are independent. For participant number j, we thus defined ‘shared anger’ faces and ‘shared fear’ faces based on a median split of the mean ratings provided by all other participants: ‘shared anger’ if ${\rm{mean}}{}_{{\rm{subj}}}[rating({\rm{face}}=i,{\rm{subj}}\ne j)]$ exceeds its median value across faces, and ‘shared fear’ otherwise.

To compute idiosyncratic associations for participant number j, we subtracted the mean rating of all the other participants (shared component of the rating) from the mean rating of participant number j, leaving the residuals $\varepsilon$ that only mirror the idiosyncratic (participant-specific) component of the ratings. Similarly to shared associations, we defined ‘idiosyncratic anger’ faces and ‘idiosyncratic fear’ faces based on a median split of the residuals $\varepsilon$: ‘idiosyncratic anger’ if $\varepsilon  > 0$, and ‘idiosyncratic fear’ otherwise.

Finally, for each participant, we performed general linear regressions to determine whether shared and idiosyncratic associations correlate with induced associations. A t-test against zero of the correlation coefficients was conducted to test for significant correlations. Induced associations did not correlate with shared associations (*T*_1,30_ = 0.32, p = 0.74, t-test against zero), nor with idiosyncratic associations (*T*_1,30_ < 0.01, p = 1, t-test against zero).

#### Emotion recognition task analyses

Repeated-measures analyses of variance (ANOVA) were performed on the proportion of correct responses in the unbiased trials ‘test’ period (removing the ‘induction’ period). Shared associations (‘shared anger’/’shared fear’) *or* idiosyncratic associations (‘idiosyncratic anger’/’idiosyncratic fear’) *or* induced associations (‘induced anger’/’induced fear’) and emotion (anger/fear) were within-participant factors.

### Model selection

We performed model-guided analyses of the behavioural data to characterise the observed influence of face-emotion associations on emotion recognition accuracy. We used Bayesian model selection based on the model evidence (estimated by a 10-fold cross-validation estimation of model log-likelihood, which penalises implicitly for model complexity without relying on particular approximations such as the Bayesian Information Criterion or the Akaike Information Criterion). We applied both fixed-effects^35,36^ and random-effects statistics^37^.

We used a simple model accounting for subject’s decisions using a psychometric model such as:

2 $${\rm{P}}({\rm{Anger}})={\rm{\Phi }}({\rm{w}}\ast {\bf{x}}+{\rm{b}})$$

where P(Anger) corresponds to the probability of judging the face as angry, Ф to the cumulative normal function, *w* to the perceptual sensitivity to the displayed emotion, **x** to the evidence in favor of anger or fear in each trial and *b* to an additive, stimulus-independent bias toward one of the two responses/emotions. We compared two models deriving from this simple model each proposing different mechanisms to account for changes in recognition accuracy based on face-emotion associations. Model 1 considers that faces associated with an emotion bias the recognition in favor of that emotion. Model 2 proposes that face-emotion associations selectively increase the sensitivity to congruent emotions. These models were used for effects related to shared, idiosyncratic and induced associations. After isolating winning models in each case, we used a full model that integrated all three types of associations changing the response bias.

Where useful, Bayes factors were computed as means to test for a critical absence of effects observed, to distinguish between the lack of sensitivity of tests and genuine absence of difference^38^.

### EEG analyses

EEG analyses were performed on a previous EEG study consisting of 24 participants that completed an emotion recognition task on morphed emotional expressions of anger and fear. EEG activity was recorded using an EEG cap of 63 sintered Ag/AgCl ring electrodes. Pre-processing of EEG data is described in details in El Zein *et al*.^10^.

The approach we use for analyzing EEG data consists in performing single-trial regressions against variables of interest, instead of averaging trials to compute evoked potentials. As the task comprised categorisation of anger and fear emotions, emotion strength was the decision variable and one variable of interest. Here we wanted to assess whether shared associations were also represented in the brain along with the decision variable, i.e. emotion strength. The strength of the parameter estimates of this regression at each time point after stimulus onset represents how much EEG activity *encodes* emotion levels or shared associations. As no ‘post-test’ rating task was used in the EEG experiment, we defined shared associations directly from participants’ behavior on the emotion recognition task using participants’ decision biases (see equation 2) when categorizing faces as angry or fearful. When computed separately for each face for all participants, the mean decision bias/criterion captures the shared component in emotion recognition. For each participant, shared associations between a face and an emotion thus corresponded to the mean emotion bias across participants for that face. We were interested in whether, in addition to encoding emotion strength, decision related brain signals encoded shared face-emotion associations. We thus looked at EEG activity at centro-parietal electrodes (CP1/CP2/CPZ/P1/P2/PZ/PO1/PO2/POZ) that encoded the decision variable^13^ and that was shown to co-vary with emotion strength at 500 ms^10^. We entered both emotion strength and shared face-emotion associations as predictors of EEG activity on these electrodes. We then looked at the parameter estimates of this regression including emotion strength and shared associations, at each time point after stimulus onset on the centro-parietal electrodes.

To look for a neural signature of both shared and idiosyncratic face-emotion associations and compare it to other types of biases, we used single-trial regressions looking at parietal activity in trials where responses were congruent or incongruent with shared, idiosyncratic (both stimulus-dependent/associative) and stimulus-independent biases.

Idiosyncratic biases were computed by using a residual approach as described in the following equation:

3 $$decision\_bias\,({\rm{face}}=i,{\rm{subj}}=j)=\beta \,\times {\mathrm{mean}}_{{\rm{subj}}}[decision\_bias({\rm{face}}=i,{\rm{subj}}\ne j)]+\varepsilon $$

with ${\rm{face}}=i$ corresponding to face number i, ${\rm{subj}}=j$ to participant number j, ${{\rm{mean}}}_{{\rm{subj}}}[\,]$ signifying a mean across participants.

For participant number j, ‘shared anger’ faces and ‘shared fear’ faces corresponded to a median split of the mean decision bias across all other participants: ‘shared anger’ if ${\rm{mean}}{}_{{\rm{subj}}}[decision\_bias({\rm{face}}=i,{\rm{subj}}\ne j)]$ exceeds its median value across all faces, and ‘shared fear’ otherwise. As for idiosyncratic biases, for participant number j, we subtracted the mean decision bias across all the other participants (shared component) from the decision bias of participant number j, leaving the residuals $\varepsilon$ that only mirror the idiosyncratic (participant-specific) component of the decision bias. ‘Idiosyncratic anger’ faces and ‘idiosyncratic fear’ faces were based on a median split of the residuals $\varepsilon$: ‘idiosyncratic anger’ if $\varepsilon  > 0$, and ‘idiosyncratic fear’ otherwise.

Stimulus independent or motor biases simply reflected the general tendency to respond ‘anger’ or ‘fear’ independently of the stimulus, i.e., the mean decision bias across all trials for each participant.

To assess whether EEG signals differed between trials with congruent and incongruent responses, across shared and idiosyncratic associations as well as stimulus-independent biases, we performed one single regression predicting EEG activity at 500 ms in centro-parietal electrodes with 3 different regressors: responses congruent or incongruent with shared associations, idiosyncratic associations and stimulus-independent biases (i.e., each of the three regressor consisted of +1 for trials where the participant’s response was congruent with the association/bias and −1 when incongruent). Positive parameter estimates from this regression signify an increased EEG activity for congruent vs incongruent response trials while negative parameter estimates signify increased activity for incongruent vs congruent response trials.

We controlled for type 1 errors that come from multiple comparisons across time points using non-parametric cluster-level statistics^39^. Finally, we applied a bootstrapping method to test for significant shifts in neural encoding latencies between conditions, using a ‘jackknifing’ procedure^40^.

## Electronic supplementary material

Supplementary Figure 1
