# Supplementary material for: Pervasive influence of idiosyncratic associative biases during facial emotion recognition
Source: Sci Rep. 2018 Jun 11;8:8804. doi: 10.1038/s41598-018-27102-z (PMC5996038; doi:10.1038/s41598-018-27102-z)
Supplement: Supplementary file 1 — Supplementary Figure 1 [file 41598_2018_27102_MOESM1_ESM.pdf]

Pervasive influence of idiosyncratic associative biases during facial emotion recognition

Marwa El Zein [1,2,*], Valentin Wyart [1,‡], and Julie Grèzes [1,‡]

[1] Laboratoire de Neurosciences Cognitives (Inserm unit 960), Département d'Etudes Cognitives, Ecole Normale Supérieure, PSL Research University, 75005, Paris, France

[2] Institute of Cognitive Neuroscience, University College London (UCL), WC1N 3AR, London, United Kingdom

* to whom correspondence should be addressed (marwaelzein@gmail.com)

‡ shared senior authorship

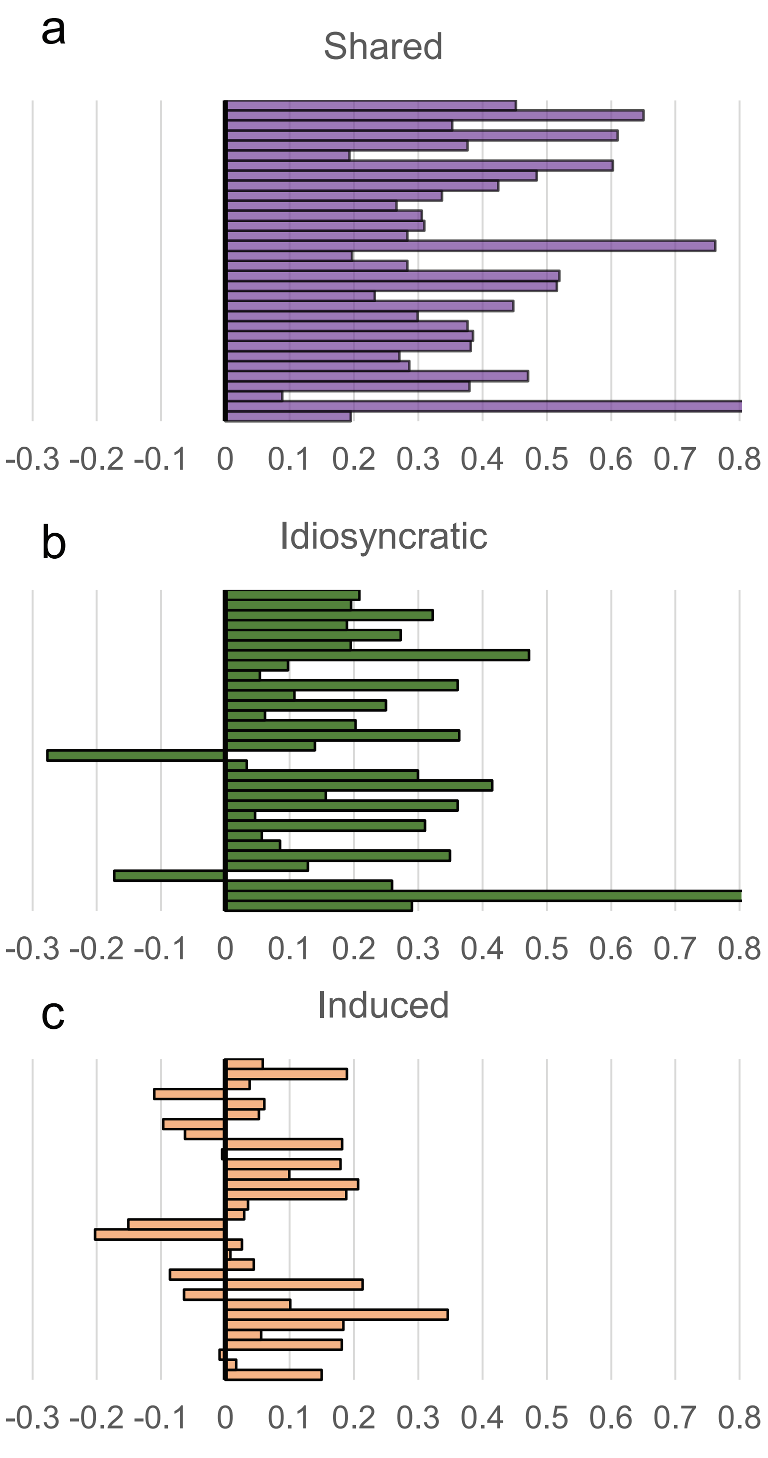

Supplementary Figure 1. Individual variability in face-emotion associations biases. Each horizontal bar represents the estimated bias for each of the 31 participants. **(a)** Individual variability for shared bias. **(b)** Individual variability for idiosyncratic bias. **(c)** Individual variability for induced bias.
